# Supplementary material for: The CBS/H2S signalling pathway regulated by the carbon repressor CreA promotes cellulose utilization in Ganoderma lucidum
Source: Commun Biol. 2024 Apr 17;7:466. doi: 10.1038/s42003-024-06180-y (PMC11024145; doi:10.1038/s42003-024-06180-y)
Supplement: Supplementary file 2 — Supplementary Information [file 42003_2024_6180_MOESM2_ESM.pdf]

Supplementary Information

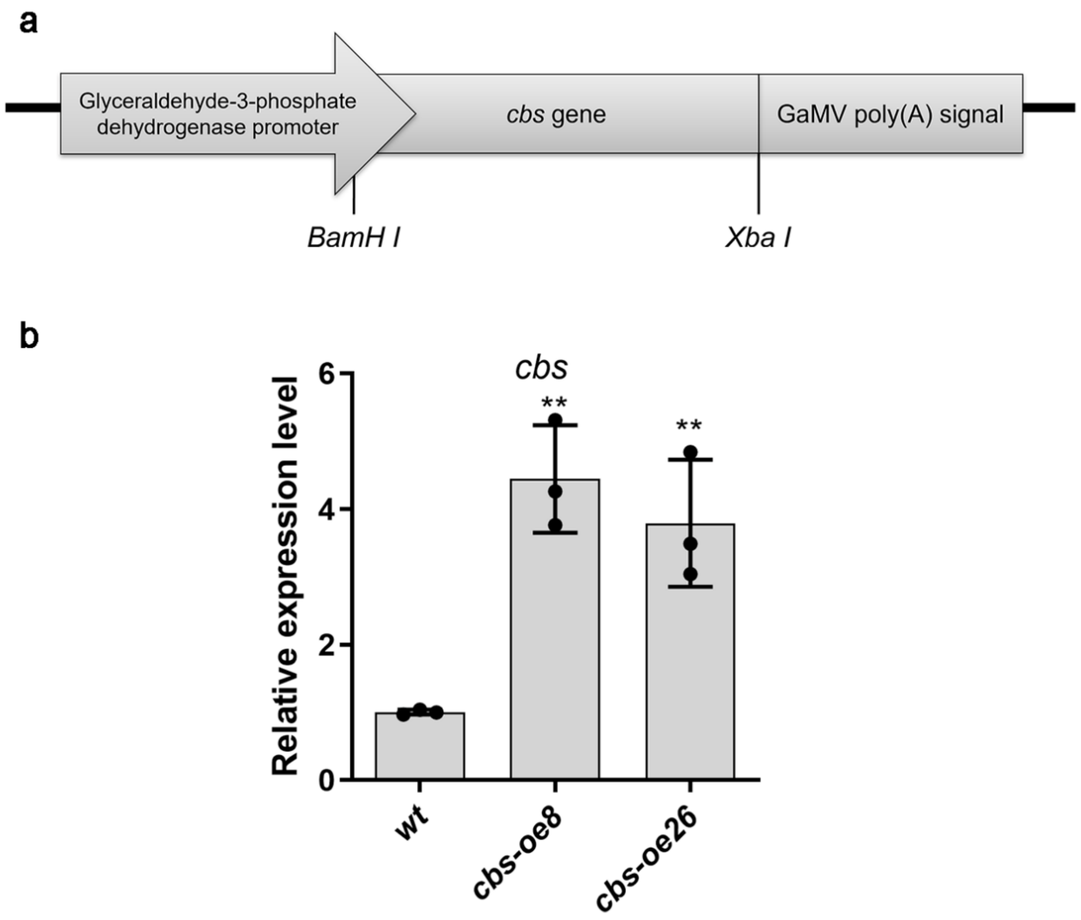

Supplementary fig. 1. Constructing and selecting of *cbs*-overexpressed strains. a Construction of the *cbs*-overexpression cassette plasmids. **b** Relative mRNA levels of *cbs* gene in *wt* and *cbs*-overexpressed strains. The expression level of *cbs* gene in the *wt* strains was arbitrarily set as the reference. The different letters indicate significant differences between the lines ("**" means $P < 0.01$, according to Student’s t test).

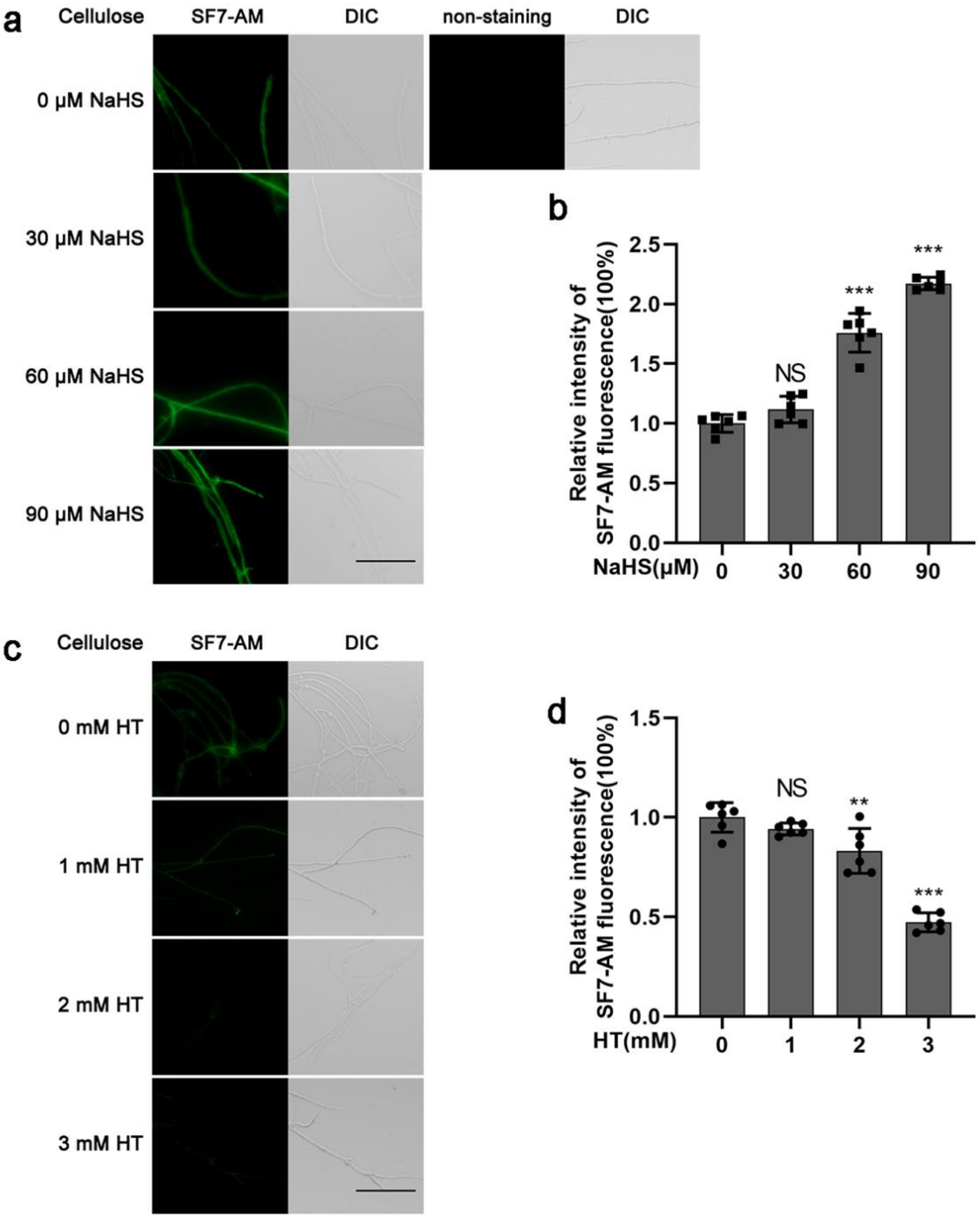

Supplementary fig. 2. The H_2S concentration in *G. lucidum*. a and b Change in the H_2S concentration was measured by SF7-AM fluorescence staining in *wt* strain, cultured under cellulose condition in the presence of sodium hydrosulfide (NaHS, H_2S donor) and hypotaurine (HT, H_2S scavenger) with different concentrations. Scale bar = 100 μm. The different letters indicate significant differences between the lines ("**" means $P < 0.01$, "***" means $P < 0.001$, according to Student's t test).

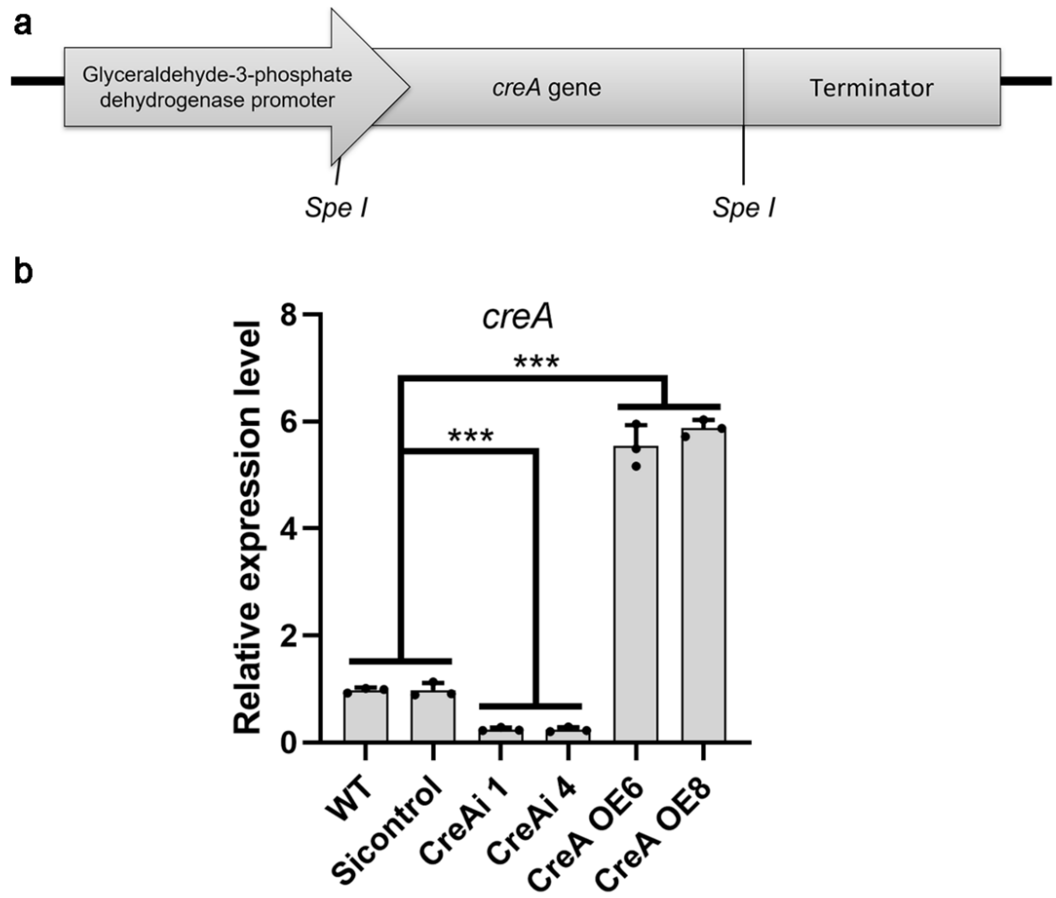

Supplementary fig. 3. Constructing and selecting of *creA*-overexpressed strains. a Construction of the *creA*-overexpression cassette plasmids. **b** Relative mRNA levels of *creA* gene in *wt*, *sicontrol*, *creA*-silenced and *creA*-overexpressed strains. The expression level of *creA* gene in the *wt* strains was arbitrarily set as the reference. The different letters indicate significant differences between the lines ("***" means $P < 0.001$, according to Student's t test).

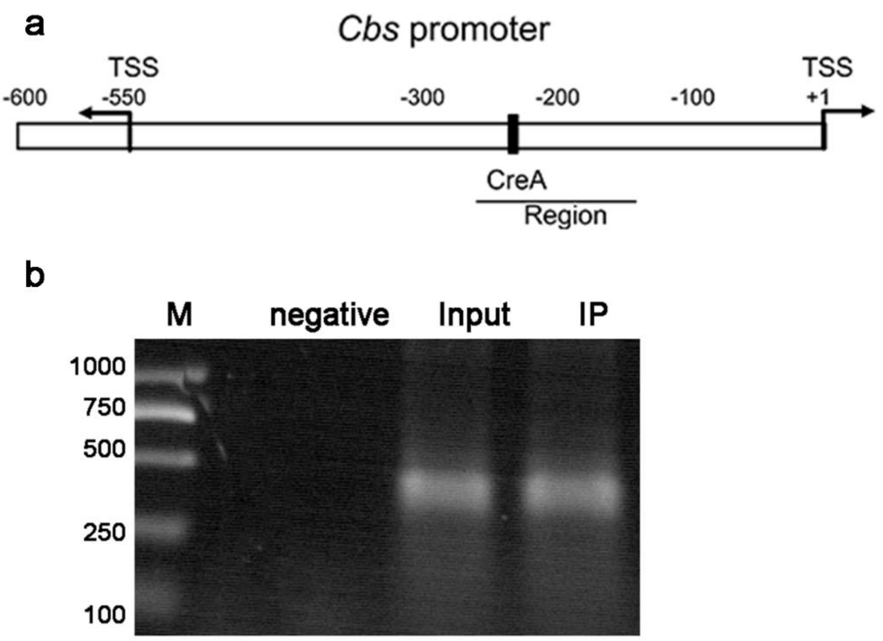

Supplementary fig. 4. Region Validation of Chromatin immunoprecipitation assay (ChIP). a The region in *cbs* promoter used ChIP-PCR and ChIP-qPCR. **b** Validation of the binding of CreA with *cbs* promoter in the region by ChIP-PCR. The sheared chromatin was immunoprecipitated with rabbit polyclonal anti-CreA antibodies (IP) and rabbit serum (negative control). Another aliquot of sheared chromatin without incubation with antibodies was prepared as input.

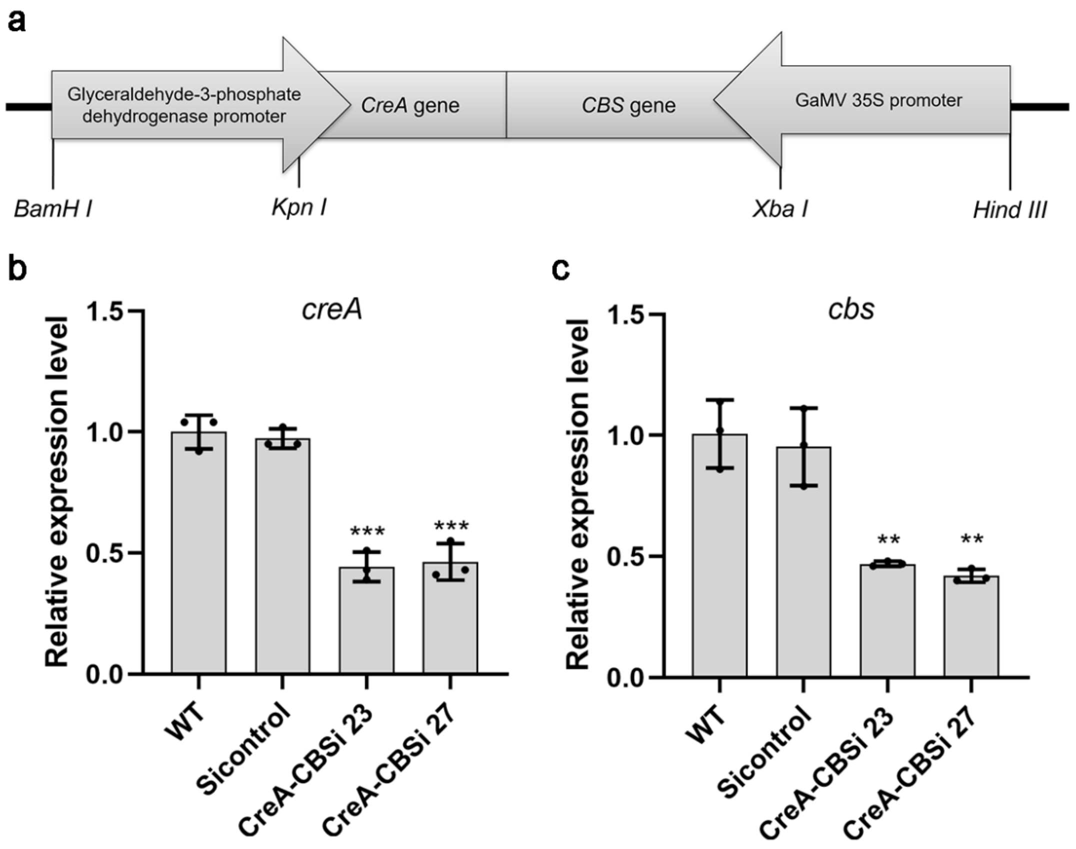

Supplementary fig. 5. Constructing and selecting of *creA*-*cbs*-silenced strains. a Construction of the *creA*-*cbs*-silence cassette plasmids. **b and c** Relative mRNA levels of *creA* or *cbs* gene. The expression level of the *creA* or *cbs* gene in the *wt* strains was arbitrarily set as the reference. The different letters indicate significant differences between the lines ("**" means $P < 0.01$, "***" means $P < 0.001$, according to Student's t test).

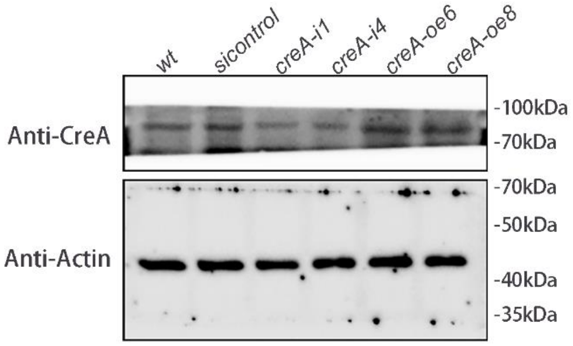

Supplementary fig. 6. Information about western blots. a Uncropped Western blots.

Supplementary tab. 1. Putative TFs directly bound to cbs promoter identified by a yeast one hybrid (Y1H) library screen.

Identifier	Description	Sequence (5′to 3′)
GL19424-G	transcription factor CreA	GGCCACGGAGACATACGACGTACCAGATTACGCTCATATGACAAGTTTGTACAAAAAAGTTGGGAGAAAGTCGTCGCCCGCCCGTACAAGTGCCCCTACCCACTTTGTGGTCGCGCGTTCAGTAGACTGGAACACCAGACCCGCCATATCCGCACGCACACTGGGGAGAAGCCTTTCGAGTGCACCTTCCCCGGTTGCGAGAAGCGCTTCTCGCGCTCCGACGAGCTCACCAGACATTCCCGCATACACAACAACCATGACACCGTGCACCACGCTGGCGCTGCAAGCTCAAGTCATGCCCACAAAACTAAGGGCAAGGCGAAGATCGACTACATAGCCGACGGCAGCATGGAGATCAGCGCTGGCAGATTCTCGACCACGCTTTCCAGTGCTGATACCGACTACGCGAGGGGAGCCGGCATGCGTGTTAAGAAGAAGGCTAGGAGCCGAGCCAACAGCGACGATGTGGGCGAATCCTACGCGCGCCCCACCGCTCTGTATTCGGCCGACCCCACAGGTTACGATTACCCCATGTCCAACCGCCGGGCCGGTCACTCCGTCAGCCACTCGCGTCCCACTCCGTCAAGTCCCAACGCTTTCTCGGCTTTGTCGAGCGTTGCTATGGAAGAGCTATACGCTCTGGAACGCGACGAGGCGCTACGGAGAGCCGAGTTCGAGGTTCGCCGCATGGAGGCGCTTCGTCGCGCGGAGTACGAGACGCGCCATGCCGATGTCCTACCCGTCCACGGCAGGCTCACGAAGAGTGCGACAAACTCGCCCATAGGCACGCCCTTTTTCTCCGCTCACGTCCCCGCTGGCGACGATGGTGGGTACTTCGGCGTTTCTAGGGATCGGGATTCCCATCCTGGGGACGATGAGCCCATCACCATCAAACAGGGGCGGAGGCGCAGTCTTGGTACCTCCAAACGCGATGTCCTGTCGATGCACCCCCATTCCTCTGGCCACGTCGGAAG<br><br>GGCGCAAAGCAAGTACATACGACGTACCAGATTACGCTCATATGACAAGTTTGTACAAAAAAGTTGGGGACATGAGGATTCGCCGTCACCAATAAGCTCGGACTCCGACTCGATGCACGCTGTGCAATCACCCCCACACGTTTCACCCACAATGGCGCATCGCCTTCCCGCTGAGGCGCCTCATCATGGACTTCCCTGCGCCGTCAAGTCTCCCGGTAGCGCGGACTTCTTCACTCCCTCGACGAGTCCCTTCCTCGGCGGTCTCCGGACGCTTAACATTCATTCGAGTGGTCCTTCGCGCGCCCCGTCGCCCTTCCGTCTTCCGCCACCCTCGCTCGACAGTCCAATCGAGGAGTACGGCTATTATGATGCTCGAAACCGCAGAATCACAGCTGCTGGCAGCCCTCCATCTGTCAGTACATTGAGTGGACGCATGGCGAAGCGCAGTAGCTCCGGCGACCTCGTGGCGTACGGCGTAGCGCACGGCGTTTCTGCGGAACACTTCGCGATGCCTCCCCCGAGCTTCCACATTCCGTACACGAGCGATAGAACGTCCATCACGTCTCTGCCGACCCCACAGTTGTCAAGCGGACCCAGCAGAAGCGGAAGCAGTCCGCGTAGCCGCACGCAGTCGCTGAACTTCGCGACCCCAGCAGCGCATCGAGTTCAAGAGCGTCGTCGCCGCCCCCGACGGGAATCTACGGA
GL28195-G	transcription factor GCN4	GGCCACTCTAGTCATACGACGTACCAGATTACGCTCATATGACAAGTTTGTACAAAAAAGTTGGAGCGTGTTCTGACGACGCTTCGCCTCGATGGCGTCCATGTCGCCGGGGTTGAGGTTGACGTCCTCCTCGAGTTCGTCCTCCTCCCCAAACGCCTGGGACCGACCACGCTTCTTCTTCAACCAGGCAGTGGGAACCTCCTTCCGCGACGTCGCTGACGGCGTGACGTACTTGCGCGGCTGGATAGGAGCCTCGAGCGGCACAAGCGCGTCTGGTGTGAGGTTCTTCCGTGTACCAGTTGGCAGGGACTTGCGGAGACGACGCGCAGGGGTCCGGGGGTTGGTAAACTGCCAGGTAGCGGCGAAGCATAAAGGGACGTAGGATCAAGTGCAGGCGTGGACGGCGAAGGCATGGTGTACAGGTCATCGAAGCTGGTGGGAGCGGGCGCGACAGAATGAGGCTTAGTCGCGTCGAGCGTGCCGTAACCGCCGCCGAAAAGCATTCCCTCGTCCATGTCGGGGAAGGTGTCAGCATCGAAGATGGCGGGGCTCGTGAGCAAACCCGCAGACAAGTCAGCGGACCCGAGCGCCGGCGTAGTGAGGAAGTCCTCGAAGGGGGAGTCCTCCATCGGCGACGTCAGGTACTCGTCGAAGGGCGATGGCGGGATCGGCGGGGGTACGCCTACGCGTTTTTTGTGAATCCCGCGCACTTTGAAACGACCATGATCGTACACACCTTGCGCACTTTGGATCGCGTCTATGATCATCTGGACGCCTTAGTACAGGA
GL26649-G	transcription factor Sko1	GGGCCGGCAGTCCATACGACGTACCAGATTACGCTCATATGACAAGTTTGTACAAAAAAGTTGGAGCACATCCTCCCGCCTGACAACGTCATCCCTCTCACCAACGACCCCTCCCCTCCCCCTCCCCGCGACAACCACAACGGCCAGAACAACAATCCCGCCTCTCAGCAGCGCCCGCCCCAGCAGCCAAAGTTTGATCTTGAGCCCAATCCCTTCGAGCAATCCTTCGCCCGCAACAAACGACTCCTCCCTCCGCACCCAGCAAAAGAGCCCCGACTCCGCCAATCGCTCCTCCGCATCCCATTCCTCCCGCGATGACTCCCGCCCCGCTTCAAAAGCCCATGACCGCGACGACAGTGCAAAGCCCACTCTCCCCCCCATAGATGCCATCAGCTCTCCCTCCGATCCATCTTACACATCGGGATTCTCCTCCACAAATCTCTCCAACTCGCTCAGGTCCGGGCCCCTGTCCCCCGCCATGCTCGCTGGCCCCCAGCCAGACAGCAACAACGATCAGCCTCTTCCCAGCTTCGATCCTACCTCCTTCCGTACTGGCCTCACCCCGCGCACTGGACTCACCCCCAGTACAGGGCTCACGCCTATCATCGGTTCCGTGTTCCCTCCTCCATCGCCTGGCACAGCGGCATGGATCAACCTCATGAACGCGAACGGTGCGCCGCCCTCCCTTTCTAATGTCGCGACGATTACCCCGACTATGATGTAAA
GL31381-G	putative transcription factor TFIIB	GGGACCGCAACATACGACGTACCAGATTACGCTCATATGACAAGTTTGTACAAAAAAGTTGGACCGTCCGTCTCATCTGCCCCGAGTGCAGAGACCCCAATCCGAACATCGTCGAGGAGTTCAGCAGCGGAGATCTAGTATGCGGCACTTGTGGGCTAGTGTTGGGCGACAGGATCGTCGACACCAGAAGTGAGTGGCGGACGTTCGCCAACGACGAGGGCGACGACCCGTCCCGTGTCGGTGCGGCCTCAGACCCTCTCATGGAAGGCATAGAGCAGCTAGACACAACCATCTCCTTCAAGGACGGTGGTTCCGGCATCGCGCGGGAACTGCAGCGTGCAGCTTCTCGTGCGAACTCCTCGCGGTCAGAACGCAACCTTCTGCAGGCGTTCCGTGACATCTCGAGCTGGTGCGATCAGTTCTCCCTCCCCAAAACTATCTCCGATATCGCGAAGCAGTTGTATAAGCGATCGGATGAGGAGAAACTGCTCCGCGGCAAGCCGCTCGAAGCAGTCATTGCGGCGTGCATCTTCATCGCGTGTCGACAAGCGCACGTTCCCCGCACGTTCCGCGAGATCTGCAACCTCACCCACGTCAGCAAGAAGGTGCTCGGACAGTGCTACAAGGCACTCGAGCAAGCGTTCAACCTCACCCCCGGCGCGTCTCAGGACCGCCACTCTATCACGCCCAGCGGCACCACCGGTGCGGAGGACCTGCTGGTGCGGTACTGCAACCACCTCGACCTGCCTCCGAGTTCCAGTCCATCTGCGCGGCCATAGA<br><br>GGGACCGCAACATACGACGTACCAGATTACGCTCATATGACAAGTTTGTACAAAAAAGTTGGACCGTCCGTCTCATCTGCCCCGAGTGCAGAGACCCCAATCCGAACATCGTCGAGGAGTTCAGCAGCGGAGACTAGTATGCGGCACTTGTGGGCTAGTGTTGGGCGACAGGATCGTCGACACCAGAAGTGAGTGGCGGACGTTCGCCAACGACGAGGGCGACGACCCGTCCCGTGTCGGTGCGGCCTCAGACCCTCTCATGGAAGGCATAGAGCAGCTAGACACAACCATCTCCTTCAAGGACGGTGGTTCCGGCATCGCGCGGGAACTGCAGCGTGCAGCTTCTCGTGCGAACTCCTCGCGGTCAGAACGCAACCTTCTGCAGGCGTTCCGTGACATCTCGAGCTGGTGCGATCAGTTCTCCCTCCCCAAAACTATCTCCGATATCGCGAAGCAGTTGTATAAGCGATCGGATGAGGAGAAACTGCTCCGCGGCAAGCCGCTCGAAGCAGTCATTGCGGCGTGCATCTTCATCGCGTGTCGACAAGCGCACGTTCCCCGCACGTTCCGCGAGATCTGCAACCTCACCCACGTCAGCAAGAAGGTGCTCGGACAGTGCTACAAGGCACTCGAGCAAGCGTTCAACCTCACCCCCGGCGCGTCTCAGGACCGCCACTCTATCACGCCCAGCGGCACCACCGGTGCGGAGGACCTGCTGGTGCGGTACTGCAACCACCTCGACCTGCCTCCGAGTTCCAGTCCATCTGCGCGGCCATAGA

GL25464-G	putative transcription factor CRZ1	GGGCACGGGGAGTCATACGACGTACCAGATTACGCTCATATGACAAGTTTGTACAAAAAAGTTGGGCCAGAC<br>CATCCCCCAAATGCTGCGCCCCATCCCCCACCAGCACGCCCCGAACACAGTGGCCTCTCTGAACCCCACGAT<br>CGGAATCCTTCTCCCCATTCCTACTACTCCCATCCTCCTCCACCTCCTGCACATGCCTCCTCCAGCCGTACCC<br>ATTACCAATCCCCTCCAAGCCCCCAGTTCCGCACCCATGCTGTCAACTGGGGTTTACCTCCCATCGGCTCCTC<br>TCAGCAGCAGCAACAGCAACTCCCACCCCCATCTCAACTTGCCGGCCCTTCGATGCCCCCTGCCCAGTTTCC<br>CCCTCCTCCAACAACATCTCGTCGTTCAAATGACGATGCCCAACGCGCGCCTCAAGCAGCGATGCTCTACGC<br>CCAATCCCAACAGCTACCTCCAATCCAAACAAACCAAGCAGGTATCGCGCCCGCCACCCGCGGACGCTCATC<br>CTCCGTGATCGCCGCCGCGTCCTCGTTCACACAAGGCGCAAATGTTCCGCGGCTCCCCCCAATCATGCAAGT<br>GGAGAAGCAGCAAGTGACCACTAGCGCGACCCAAGCAGCGAGCGCGAGTCGGCGGCGCAACGAGGCCAAC<br>TTCGTTTGCCCGGTCCCCGGCTGCGGGAGCACGTTTACTCGGCGTTTCAACCTGAGAGGCCACCTCCGCTCC<br>CACACCGCCGGATTAGCGATCCGAT
GL25297-G	putative transcription factor XBP1	GGGCACGGGGAGTCATACGACGTACCAGATTACGCTCATATGACAAGTTTGTACAAAAAAGTTGACTCCGAGC<br>CTCAGTCCCCCGACTCACATACCTCGGACTTGGCAGGTCCTCCACGGAAGCGTGCCCGCTCTGATGTTACCC<br>CGGAGGAGCGTAGAGAGGCACGCGCACACCGCAACCGTATCGCTGCACAGAACTCCCGCGACCGCCGAAAG<br>GCGCAGTTCACGCATCTCGAGCGCCGCGTCCAGGAGCTCGAGGAGGAGAACCGTCAGCTCCGTGCGGGCAT<br>GGATGTCCCAAATGTCGCATTCCCACGAACAGAAACACGATGAGCGCGAGAAGGATAGGGCCCGAGACCAG<br>GAGAATGAGGAGCTCCGGCAGAGAATCAAGACCCTGGAGACCGGGTGGGAAGCCGTCATGAAAGCCCTCAC<br>CGCCTCCTTCCCCTTAACGTCCCCTCTGCCCCCTTCCTCCTCCTCTTCGTCGTCGCCCTCAGCAACAGTTACG<br>TCTGGTCACCCTCCCACAACTACGTTCCCCGTATTTGTGCCCCCATCCCCTGTCTTCCCCATCTCCCCCGCAC<br>CATCAAACCACTCTTCCGGCTCCAGCACTCTCTTTGACTCTGACGACTTCGATTCCACTCGCCACCTGCACGG<br>GTGGCGACCACCGATGCCCCTCGTTTATCATCGGTGTCCCTGCAGCGGCTGGAGCTCAACCTCCCCACACTC<br>ACCTTTCCGCCGACCATCCAGATCGGTGATGGCGCGCCCTCGGCCGTGGACGAAGTCGCGATGGACGACCT<br>CTTCCGAGAGATCCTCGCGCCTTCTCCTGTGTCGCCGCAGGCTCCTCTGCCTGCTGATGCGCCCTCCCGGTC<br>ACGATCCCAGTCGTTCTCCACCGAAATCCAGCTGGCCCCGGCACCTTCCCCGGCGCCGATGACCCGGCCCC<br>TGCCTTCACTACCGGAGATCGACTGGGAGAGATCTATCCATCGTA
GL23559-G	putative transcription factor MCM1	GGCACTCCAGACATACGACGTACCAGATTACGCTCATATGACAAGTTTGTACAAAAAAGTTGGTGTCCAACAA<br>GCGCGCCCGCCACGAACTCGACCAGCCCGCCGTCCCCAACGACGACGGCTTCGTCCACGACCCCGACGCC<br>GCAGAGTCTGGCCAGGACGACGACGACGATGAAGACAAGCCCAAGTCGGACAAGAAGGCAGGTCGCAGGGA<br>TCGAGTTCATCCAGGACAAGTCCAGACGTCATATCACCTTCTCAAAACGAAAGGCGGGCATCATGAAGAAGGC<br>GTACGAGCTCTCCACCCTCACCGGCACCCAGGTGCTCCTCCTCGTCGTGTCCGAGACTGGCCTCGTCTACAC<br>TTTCACCACTGCCAAGCTCCAGCCCCTCGTCACCCAGCCAGAGGGCAAGAACCTCATCCAGGCATGTCTCAA<br>CGCGCCCCACGGCCAGCTCCCCTCGTCCATGCCCGTCGGTACTCCCATCAACCGCACCTCCATGCCCATGCA<br>GCACCCGCCCAACGTCCCGGGCGGCCTCGCTCGGCTCCGCCCCCAGCTCCAAGGAGGATGATCAGCAAGAG<br>GAGAGTGAGGATGCCCCGGTACCAGGCGACGGTGGTCGCAACCCTCGTAAGCGCCGTCGCGCGTCCTCGTC<br>CGTAAACCAACCCCGCGGTAACAATTCTCCGGCTGTGTCCGGTTCGTCTGCCGGCGCACCTAGCCAGTCACC<br>GCACGCTCGTCCAGGTCAAATCCCTCCCGGTGCTCCCCCAATCTCCATCCCCGGCGTCGCACCCCAGATGCC<br>CCTCGAAAGCTCTCCGCGTCAAGGCCAGGCTGCGCCGCCACCCACAAGTCCCAACTATGCCCAACCGGCAG<br>GTGGCTACCCGCCCGCGCCTGCGAGTGGGGACCCGAACATGTACGCGCACAGTGGCTACCCCGGCGCGCC<br>TCCGCCTCAGCAATACGCGACGTTCGGGCACGACGGGGCAGCGCAATCGGCCGTCAACCATATCGTGAGT
GL25906-G	ribonuclease HII	GGCGCTCCAGACATACGACGTACCAGATTACGCTCATATGACAAGTTTGTACAAAAAAGTTGGATGCCAGCAT<br>TCCGTCTGTCCCAGACCCCTCAATCCCCCTCACCGAGTCCTACACATACCATTCGCCAACTCCCACTGCTCCC<br>GGACCGTATTTTCTTGGCGTAGACGAAGCGGGAAGAGGCCCTGTGCTAGGACCTCTCGTATATGGCGTCGCA<br>TACTGCCCCCTCGCTTACAAGGAGGAGCTCGAAGGGCTCGGGTTCACCGATTCGAAAACGCTGACCGCGAAG<br>GTGCGCTCTTCGCTTCTACAAACCTTGGGGTCCGACCCGGCCAACCTCGGTTGGTCAGTCCGTGTCTTGAGC<br>CCGAAAGCCATCTCCGCCGGCATGCTCAAACGACCCCCAACGAACCTCAACCAGCAATCGCAGAACGCGACC<br>ATCCTGCTTATCCGCGAGGTCCTCGCCAAAGGCATCCAGCTCTCCGAGGTGTACGTCGACGCGCTCGGGAAG<br>ACGACGACTTACCAGGCCTACCTCTCCTCCCTCTTCCCCGACATCTCCTTCACGGTCCAGGCCAAGGCGGAC<br>CTACCTTCAAGATCGACGGCGCCGCTAGTGTCGCTGCCAAGGTCACGCGCGACGCGTGGATCGAGGGCTGG<br>GTGTACGAAGAGAATACCGCCACTCACGAACTCATCCAATCCAAATTCTTTCGCAACCCGTCATCGGCACCAG<br>CGGAGAAGGAGAAGGCGGGGAAGAAGTCCAGAAAATGGGTGGAGGAGACGGGCAGCGGGTACCCATCGGA<br>TCCTAAGACGCAAGCTTGGATCAAGGGATCGCTGGAACCTACTTTTGGATACCCTTCGTTCGCCCGATTCTCC<br>TGGGCGACCATCAAGGTCGCTCTGGAAAAAGACGGACATGCAGTGGAATGGATCGACGACGGTCAAGCATCT<br>CTAATGCAAGCCTTCACCACCGCAACTAGTAGT
GL25876-G	5'-3' exoribonuclease 2	GGCACGCAGACATACGACGTACCAGATTACGCTCATATGACAAGTTTGTACAAAAAAGTTGGATTAATCTCAAC<br>TTTCCTCCCCCAGGCCAGGACGGTGCTCCTGCAAATGCCCAAGGACGCCCCGAACACGCTCGTCCGAGGCA<br>TGAGTCCTGGGCCCGTTCGAGTGGTTCGGCACCCATGCCCCAGCCTCAACACTCTTCAGCGATGCCACCCCC<br>ACACGACCCGCATTCAAAGCACCAGCACCACCAGTACGCGCCCCCACCCAAGTCTGACCCACCATACGCGCA<br>GCCCGCTCCCTCGCCGCAGCAACCCACACAGGGTGTCCCCACACACAACAGTGCCAGTGCGGGTGCACCGC<br>GTCCCGAGAACCCTGCGGACGCTTCCTCCAAAAGGTCCCGTTCGCAGCAAGGCGTGAGCGTCTCGCCTGGA<br>CGTTCCCCTCACACCGTGTACCCCTCGTATCCCACTCAGCCGCCCCAGACGGCATACCACCAGGGCCCGCCT<br>TCCCAGGGCCCTCCGCCCCCGCCTCCAGAGCATCTCCAGCAGCCTCCCCATTACCCTCATCAGCCTACGCCC<br>GCGGGGTATGCCCCGTACCCCCAGGCAATGCCGCGAGGGCATTATGGTATGCCTCCGCCACCCCCTCCTGC<br>CCACCACCAAGGCCAGCCACCGCCCAGCCCGTATGCGCCGCCTGCCGAGCACTGGCAGCTGCTCAGCCCCA<br>GCCGCCACCGCAACAACAATTCAATGGCTATACACGCACCACTCCGTTGGTGCCGGCACCTGTAGAGGCACG<br>GGCTCCAGCTCCTCCCGTTCCAAGTCCAGCAGATGTAGAACGGGCTAATGCGCGTCATCAGGTGATTGCCGA<br>GATCTTGGATCACTGTAACGTCTTGTACAGCTTTGCGAATCATTACGCCGGCATGACTCGCCACAACCCACAA<br>CTGCAACCCGCGCCGCATGAGATTGAGAAGATGAACTTCCACGCCTCGATGCGCCTTCTCATTCGATGC
GL29208-G	putative transcription factor	GGGCAAGGCAGTACATACGACGTACCAGATTACGCTCATATGACAAGTTTGTACAAAAAAGTTGGAGCGAATG<br>GCCAGTACCCGATGCCCATGATGGCCCCGATGGCCCAGGACGAGCAGGGCAAGAAGCGCAAGTCGCGCGTT<br>GAGGACGGAGAGGGGAAGAGGAGAGCGAAGAAGCCCAAGGATGTCAACGCTCCGAAGCGTCCCGCCTCCTC<br>CTACCTCTTGTTCCAAAATGACGTCCGCAACGAGCTCAAGCAGAAGAACCCGGGCATGCGCAACAACGAACT<br>GTTGAGCACGATCTCCAAGCTGTGGTCGGATATGCCGCAAGCTCAGAAAGATGTATACGAGGCCCGTAACAA<br>GGAACTGAAGGATCAATGGCGAGCTAACAAGGCTGCCTACGAGTCTGGCAGGCTGGGGCATCTACGTTGCCT<br>GTCGCGGTCAGCGCTCCTGTTTCCGTCCCAGTTGCCCCTGCCCCCATCGTCAAAGCTGTTCCCACCGTCGCC<br>CCAGTGGCCGAGGAAGAGTCTTCTGAGGAGGAGTCCGCAGCTGAGGAGGATGAAGATTCGGAGGAGTCAAG<br>CTCGGAGGATGAGGATGATGAGGTAGCACCTCCAGCGAAGAAGTCGAAGAAGGAGACTGCTGTAGTAGCTAG<br>CTTAGCTGGC

GL29043-G	putative transcription factor	GGGACCGCAGACATACGACGTACCAGATTACGCTCATATGACAAGTTTGTACAAAAAAGTTGGAGTTACAATCCTGAGCACTACCCTGCCTATGACGAACAATTCCGTAGGGTTGGTATGGAGCATGCACGCTTCGGCACAGACCCCGGCATACCAGACCAAGCATACTACCGCCCACGGACGCACACATACCCCCCTGTGGGTGCTACCCCGCTTCCGCTGGGAATGCCATCGCCTGAGCCGGCTCATCTGTCAAATTTCCCGTCACCCGAGCTTGACCTGCTCCCCATGCACTCTGATGAGCCCCCCTTCAATCGGGACATGCGCCAGGACCACGTTATCCCCGGACAGACGCGTATTATCTCCCATCCCCGGTCGCGCCACAGACCCAAGATACGCAGGCTTACGCTAGTACCTCGAGCTCCCCCCTCCGTTCTGGCGACGACTCCTACATCCCCACTTCTGATGCCGACATTCGACGGATTCTTGGGCTCAGTCCCGATCAGGAACTGAGTCTCAACGCCCTCGCGGACCCACCTCCCGGTCAACGTCCTGGTCAATCCATCCCTACTCTCTCTCAGCTCGCCATCCTTGGCAGCCAGAACAAGCGGTTGACGCTTCAGGAAATCTACCAAGCACTCGAGGTAGGTTCGAGTGGTTCGCTCAAAATAGGGACGATAAGTCGTGGCAGAACTCCATTCGGCACAACCTTTCCCTCTACAAATGCTTCAGACGGATCCAGAAGCCCATCACGGAGCCGGGCAAAGGCAGCTACTGGGTCGTCGACTACTCGGATGGCTCGGGCACGAAGCGTCCTCGTAAACGCAACAAGCGTCCGACCAAGGCCCAGTTGCGTGCTCAGGCTGCAGATGAAGCGCAGGAGATGCAGGCGCAAGCTCAGGACGATGTTGAGAGCTCCCCGGAGGAAGACGAAGGCTTCCCCTCGCCTGCCCAGTCCCAGGACATGTTTGTCGACCCCATGCTCCAATCGTGTCAGTCGTAGCATTAGCG
GL27308-G	putative transcription factor	GGGCGATGCAGACATACGACGTACCAGATTACGCTCATATGACAAGTTTGTACAAAAAAGTTGGAGTCTCTTCAAGAAGGCCTACGAGCTCGGCGTGCTTTGCTCCGTCGATGTCGCCGTTATCATCTTTGGACATCACGCGAAGCTCTACCAGTACTGCTCGACGGATGTCAACGGCATGGTCCAGCGTCACATCCGCCCTGCCCAGTTTGACGGTGAGCGGGATACCAAAGGCCCTCCTGACTTCAACAACAGCGCGGGCAAGGCCACAGAGGAGCGGCCAT
		GGCAATACAGACATACGACGTACCAGATTACGCTCATATGACAAGTTTGTACAAAAAAGTTGGCGGCGCAAGAACGGTCTCTTCAAGAAGGCCTACGTTGCTCGGCGTGCTTTGCTCCGTCGATGTCGCCGTTATCATCTTTGGACATCACGCGAAGCTCTACCAGTACTGCTCGACGGATGTCAACGGCATGGTCCAGCGTCACATCCGCCCTGCCCAGTTTGACGGTGAGCGGGATACCAAAGGCCCTCCTGACTTCAACAACAGCGCGGGCAAGGCCACAGAGGAGGCGGACGATGACGACGAAGCGGACGACGAGGAGAGCTCACAAAAGCGCCGGGATAGCAGCTCTTCAAAAGCACCTCCGAAAGTGAAATCAGAAAACAACTCAAGCAGCATCGTCCCAACGCCCCGGCCCCCCTTACATACGTGTACG
GL15296-G	putative transcription factor	GGCCACTCTAGTCATACGACGTACCAGATTACGCTCATATGACAAGTTTGTACAAAAAAGTTGGAAGTCGGCGACAAGAAGTACGCCTGCGAGACCTGCATCAAGGGCCACCGCTCCTCCTCCTGCAAACACACCGATCGTCCGCTGTTCGAGATTAAGAAGAAGGGTCGCCCTATAACCCAGTGCGAACACTGCCGTGAGCTTCGGAAAACCCGCCAAATTCACGTGAAATGCGTCTGCGAGAGCAAGGAGATCGCCGAAGAGGGGCTCGGCGCGACGGGCAGCAAAGGAACAAAGTTGCCGGCCCGCGGCGCGTTTCCCAGTGGTTTGCCGGAGGAGCTGCTGGAAGCGTCGGTTGCGTCTCAGCCCTTCTCCGAAGGTTCTGACTCGGAGCAAAGCACTAGCGAGCGCCGCGGCTCTGAAGCTCGCCCCATCGCACCCTCGCCGAATGAAGCTCTATCTGAACCCGCTGCGCTTGTCGCTCAAGCCCACGCAGCGGGCAATCGTCCGGTTCTGCCCAAACCCCCCACTGAACGTCCGGACTCACCTTCTCGCGCGTCACATAACCCCTCTGTCTCCCAAAGTAGCCGTCTCCCCAGCCACGGCCAGTCATTCTACAGTCCCTATGGCCGCGCTTACGACTACTCCCACGCTACGGGCTATACGACTTTCGGTGACGGCAATGCTTCCAGCCTGCAGCACCTGCTCAACGCTGCCCCCAATTCCACCACCCCTTTCGACACCTGGTCGCTCAGTGCCGATAACGCGGGTTTCCCAATCTCACCTGCATCCGCCTCTCCCCCTGTTTGCGGCTGCGGCACCTCCTATCTGTAGCTGATAGCT

Supplementary tab. 2 Oligonucleotide primers used.

Primer	Sequence (5′to 3′)	Description	gene NCBI accession number
lcd1-RT-F	GCTCGGTGGTGAGAAGG	Detects the *lcd1* expression	AUN37957
lcd1-RT-R	TGGGTAGCGGCAACTGG		
lcd2-RT-F	AGAATCGTAGCCGTCATCG	Detects the *lcd2* expression	AUN37958
lcd2-RT-R	GCAGTTGGAGACCCAGAAGT		
cs1-RT-F	TCAGTGGATGGCTTCGTTG	Detects the *cs1* expression	AUN37951
cs1-RT-R	CGTCCCTCCAGGCTTCA		
cs2-RT-F	AACCGTCTCACGCATAAC	Detects the *cs2* expression	AUN37952
cs2-RT-R	CTGGAACCCAAGAACAAT		
cse1-RT-F	GCTTAGGTGGCGTGGAGA	Detects the *cse1* expression	AUN37953
cse1-RT-R	CAGACAGCCGGATGAGGT		
cse2-RT-F	TCCGAACAGGGAAGATGG	Detects the *cse2* expression	AUN37954
cse2-RT-R	TTGCCGCGTCCTAATGC		
cse3-RT-F	CCCTATCATCCGCATCG	Detects the *cse3* expression	AUN37955
cse3-RT-R	CCAGCCTTGTGGACCTT		
cse4-RT-F	GACCACCACCGTTACCCA	Detects the *cse4* expression	AUN37956
cse4-RT-R	GCTTCTCGCCTTCTTCGT		
cbs-RT-F	TACACCGCAAATCATCG	Detects the *cbs* expression	AUN37950
cbs-RT-R	CCTTTCCCACTAACAACC		
3-mst-RT-F	GTTCTAAGGGCCGTTTC	Detects the *3-mst* expression	AUN37948
3-mst-RT-R	CTCTGGGATGTTGTGGG		
creA-RT-F	GGCACTCCGTCTGGTCAC	Detects the *creA* expression	MH145352
creA-RT-R	CGCCATTGTGGGTGAAA		
