# Supplementary material for: The CBS/H2S signalling pathway regulated by the carbon repressor CreA promotes cellulose utilization in Ganoderma lucidum
Source: Commun Biol. 2024 Apr 17;7:466. doi: 10.1038/s42003-024-06180-y (PMC11024145; doi:10.1038/s42003-024-06180-y)
Supplement: Supplementary file 3 — Description of Additional Supplementary Files [file 42003_2024_6180_MOESM3_ESM.docx]

**Description of Additional Supplementary Files**

**File name:** Supplementary Data 1
**Description:** The source data behind the graphs in the paper can be found in Supplementary Data 1.
