# Supplementary material for: The CBS/H2S signalling pathway regulated by the carbon repressor CreA promotes cellulose utilization in Ganoderma lucidum
Source: Commun Biol. 2024 Apr 17;7:466. doi: 10.1038/s42003-024-06180-y (PMC11024145; doi:10.1038/s42003-024-06180-y)
Supplement: Supplementary file 5 — Reporting Summary [file 42003_2024_6180_MOESM5_ESM.pdf]

nature portfolio

Corresponding author(s): Mingwen Zhao
Last updated by author(s): Feb 19, 2024

Reporting Summary

Nature Portfolio wishes to improve the reproducibility of the work that we publish. This form provides structure for consistency and transparency in reporting. For further information on Nature Portfolio policies, see our Editorial Policies and the Editorial Policy Checklist.

Statistics

For all statistical analyses, confirm that the following items are present in the figure legend, table legend, main text, or Methods section.

n/a	Confirmed	
☐	☒	The exact sample size (*n*) for each experimental group/condition, given as a discrete number and unit of measurement
☐	☒	A statement on whether measurements were taken from distinct samples or whether the same sample was measured repeatedly
☐	☒	The statistical test(s) used AND whether they are one- or two-sided<br>*Only common tests should be described solely by name; describe more complex techniques in the Methods section.*
☒	☐	A description of all covariates tested
☐	☒	A description of any assumptions or corrections, such as tests of normality and adjustment for multiple comparisons
☒	☐	A full description of the statistical parameters including central tendency (e.g. means) or other basic estimates (e.g. regression coefficient) AND variation (e.g. standard deviation) or associated estimates of uncertainty (e.g. confidence intervals)
☐	☒	For null hypothesis testing, the test statistic (e.g. *F*, *t*, *r*) with confidence intervals, effect sizes, degrees of freedom and *P* value noted<br>*Give P values as exact values whenever suitable.*
☒	☐	For Bayesian analysis, information on the choice of priors and Markov chain Monte Carlo settings
☒	☐	For hierarchical and complex designs, identification of the appropriate level for tests and full reporting of outcomes
☒	☐	Estimates of effect sizes (e.g. Cohen's *d*, Pearson's *r*), indicating how they were calculated

Our web collection on statistics for biologists contains articles on many of the points above.

Software and code

Policy information about availability of computer code

Data collection	We have provided a description of all source used to collect the data in this study and specified the version of software used in this study.
Data analysis	We have provided a description of the version of software used to data analysis in this study.

For manuscripts utilizing custom algorithms or software that are central to the research but not yet described in published literature, software must be made available to editors and reviewers. We strongly encourage code deposition in a community repository (e.g. GitHub). See the Nature Portfolio guidelines for submitting code & software for further information.

Data

Policy information about availability of data

All manuscripts must include a data availability statement. This statement should provide the following information, where applicable:

- Accession codes, unique identifiers, or web links for publicly available datasets
- A description of any restrictions on data availability
- For clinical datasets or third party data, please ensure that the statement adheres to our policy

All data presented in this manuscript are available from corresponding author upon reasonable request.

Human research participants

Policy information about studies involving human research participants and Sex and Gender in Research.

Reporting on sex and gender	The information about sex and gender has not been collected as it did not involve Human research participants.
Population characteristics	The information about population characteristics has not been collected as it did not involve Human research participants.
Recruitment	No information about recruitment as it did not involve Human research participants.
Ethics oversight	No information about ethics oversight as it did not involve Human research participants.

Note that full information on the approval of the study protocol must also be provided in the manuscript.

Field-specific reporting

Please select the one below that is the best fit for your research. If you are not sure, read the appropriate sections before making your selection.

☒ Life sciences ☐ Behavioural & social sciences ☐ Ecological, evolutionary & environmental sciences

For a reference copy of the document with all sections, see nature.com/documents/nr-reporting-summary-flat.pdf

Life sciences study design

All studies must disclose on these points even when the disclosure is negative.

Sample size	No statistical method were used to determine sample size.
Data exclusions	No data were excluded from this research.
Replication	Data presented were repeated for at least three times independent experiments.
Randomization	Randomization did not apply to this research as it did not involve animal experiments and clinical trials.
Blinding	Blinding did not apply to this research as it did not involve animal experiments and clinical trials.

Reporting for specific materials, systems and methods

We require information from authors about some types of materials, experimental systems and methods used in many studies. Here, indicate whether each material, system or method listed is relevant to your study. If you are not sure if a list item applies to your research, read the appropriate section before selecting a response.

Materials & experimental systems

n/a	Involved in the study	
☐	☒	Antibodies
☒	☐	Eukaryotic cell lines
☒	☐	Palaeontology and archaeology
☒	☐	Animals and other organisms
☒	☐	Clinical data
☒	☐	Dual use research of concern

Methods

n/a	Involved in the study	
☒	☐	ChIP-seq
☒	☐	Flow cytometry
☒	☐	MRI-based neuroimaging

Antibodies

Antibodies used	The primary antibodies used to detect specific proteins in this report were anti-CreA (1:2,000, rabbit polyclonal, self-preparatied in previous work: PMID: 31608522), anti-Actin (1:2,000, mouse; CMCTAG) and anti-Histone3 (1:2000, AT0005, CMCTAG).
Validation	CreA antibodies were validated in previous work: PMID: 31608522. All other antibodies were extensively validated in previously published papers and by suppliers.
